# Targeting FBXO44/SUV39H1 elicits tumor cell-specific DNA replication stress and viral mimicry

**DOI:** 10.15698/cst2021.03.245

**Published:** 2021-02-18

**Authors:** Jia Z. Shen, Charles Spruck

**Affiliations:** 1 Tumor Initiation and Maintenance Program, NCI-Designated Cancer Center, Sanford Burnham Prebys Medical Discovery Institute, La Jolla, CA 92037, USA.

**Keywords:** FBXO44, SUV39H1, replication stress, viral mimicry, immunotherapy

## Abstract

Repetitive elements (REs) are normally transcriptionally silenced in somatic cells by repressive epigenetic modifications, which are thought to include DNA methylation and histone modifications such as deacetylation, H3K9me3, and H4K20me3. Although, it is unclear how RE silencing is maintained through DNA replication cycles in rapidly growing cancer cells. On the other hand, the reactivation of endogenous retroelements beyond a threshold level of tolerance in cancer cells, such as by treatment with DNA demethylating agents or HDAC or LSD1 inhibitors, can induce viral mimicry responses that augment certain cancer therapies, including immunotherapy. However, these agents can also affect normal cells presenting obvious side effects. Therefore, uncovering cancer cell-specific RE silencing mechanisms could provide a basis for the development of a new generation of cancer immunotherapy drugs. In our study (Shen *et al.* (2020), Cell, doi: 10.1016/j.cell.2020.11.042), through a high-content RNAi screen we identified FBXO44 as a key regulator of H3K9me3-mediated transcriptional silencing of REs in cancer cells. Inhibition of FBXO44 or its co-factor SUV39H1 stimulated antiviral pathways and interferon (IFN) signaling and induced replication stress and DNA double-strand breaks (DSBs) in cancer cells, leading to restricted tumor growth and synergy with anti-PD-1 therapy (Figure 1).

**Figure 1:**
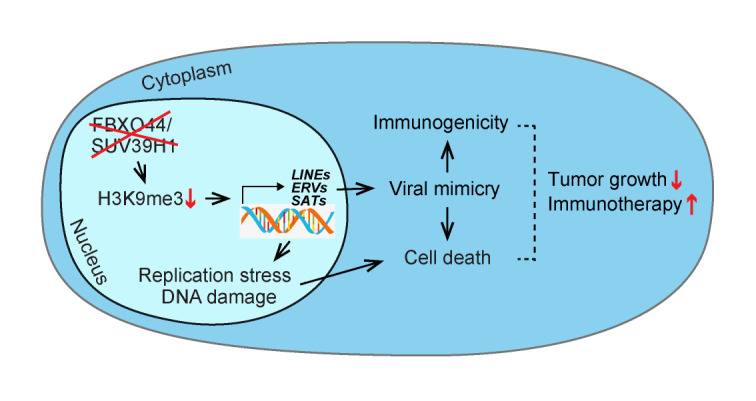
FIGURE 1: Graphical representation of this study. FBXO44/SUV39H1 targeting activates REs that elicit DNA replication stress and viral mimicry in cancer cells, leading to tumor growth arrest and enhanced immunotherapy response.

FBXO44/SUV39H1 targeting activates REs that elicit DNA replication stress and viral mimicry in cancer cells, leading to tumor growth arrest and enhanced immunotherapy response.

Approximately 45% of the human genome is composed of REs, which include interspersed long-terminal repeat (LTR)-based endogenous retroviruses (ERVs), non-LTR-based short- and long-interspersed nuclear element (SINE and LINE) retrotransposons, and tandemly arrayed satellite repeats. These sequences are normally transcriptionally silenced in somatic cells, maintained in heterochromatic regions characterized by repressive DNA methylation and histone modifications, such as deacetylation, H3K9me3, and H4K20me3. Deregulation of REs has been implicated in human aging and several diseases, including cancer. However, the detailed mechanism underlying the maintenance of repressive epigenetic modifications at REs, especially in relation to DNA replication, has remained elusive.

Previous studies demonstrated that DNA demethylating agents and HDAC or LSD1 inhibitors can induce viral mimicry in cancer cells by reactivating endogenous retroelements beyond a threshold level of tolerance, leading to antitumor activity and enhanced immunotherapy response in preclinical models. Mechanistically, the induced expression of endogenous retroelements can promote the accumulation of double-stranded (ds)RNA and dsDNA replication intermediates in the cytoplasm of cells, where they are recognized by pattern recognition receptors such as RIG-I/MDA5 and cGAS that activate adaptor proteins MAVS and STING, respectively. Activation of these intracellular antiviral pathways stimulates IRF3/IRF7-dependent IFN signaling, thus mimicking virally-infected cells. However, DNA demethylating agents and HDAC inhibitors can also target the cells of normal tissue, limiting their application for cancer therapy due to safety considerations. Hence, there is an urgent need to develop novel tumor cell-selective viral mimicry-inducing strategies for cancer treatment.

In our recent study, we reported that the F-box protein FBXO44 plays an essential role in H3K9me3-mediated transcriptional silencing of REs in cancer cells and this process could provide a therapeutic opportunity for cancer treatment. First, we performed a high-content RNAi screen to identify key regulators of repressive H3K9me3 modifications and DNA replication stress in a panel of cancer cell lines of diverse tissue origin. The results of the screen identified the largely uncharacterized protein FBXO44, whose inhibition decreased H3K9me3 levels at REs leading to their transcriptional activation. ChIP-seq analysis revealed that FBXO44 selectively interacted with REs and heterochromatin, and co-localized with repressive H3K9me3 modifications. Protein mass spectrometry and biochemical and molecular experiments showed that FBXO44 forms a repressive epigenetic complex that includes H3K9me3 methyltransferase SUV39H1, ubiquitin ligase CRL4^RBBP4/7^, and nucleosome deacetylase and chromatin-remodeling complex Mi-2/NuRD, at the replication fork in the cancer cells. Specifically, complex assembly at the replication fork was found dependent on FBXO44's ability to bind H3K9me3-modified nucleosomes, presumably targeting SUV39H1 to modify adjacent newly synthesized nucleosomes to promote the rapid silencing of REs post-DNA replication.

Further studies demonstrated that FBXO44/SUV39H1 inhibition in cancer cells transcriptionally activated various RE subtypes, including satellite repeats, ERVs, and LINE-1, resulting in the accumulation of cytosolic dsRNA and dsDNA replication intermediates and activation of RIG-I/MDA5-MAVS and cGAS-STING antiviral pathways. FBXO44 inhibition also promoted DSBs and genomic instability in cancer cells, as indicated by cGAS^+^ γH2AX^+^ micronuclei. RNA-seq and molecular analysis showed that FBXO44 inhibition activated IFN signaling, enhanced antigen presentation, and stimulated various cytokines and ligands that function to recruit cytotoxic T and natural killer (NK) cells, thereby enhancing tumor cell immunogenicity. Targeting FBXO44 by genetic knockdown or pharmacologic inhibition of SUV39H1 restrained the proliferation of breast, lung, colon, and glioblastoma cancer cells *in vitro* and tumor growth in immunodeficient mice. In contrast, targeting FBXO44/SUV39H1 had no effect on the viability of normal cells, including primary human mammary epithelial cells (HMEC) and astrocytes. Further mechanistic studies revealed that H3K9me3 modifications and FBXO44/SUV39H1 were present at REs in tumor cells but not normal cells, suggesting a therapeutic window for cancer treatment.

Given that FBXO44/SUV39H1 inhibition enhanced cancer cell immunogenicity, we next examined if it could modulate antitumor immune response using a preclinical mouse breast cancer model. FBXO44/SUV39H1 inhibition stimulated the intratumoral infiltration of cytotoxic CD8^+^ T and NK cells. Furthermore, SUV39H1 inhibitor F5446 synergized with anti-PD-1 immunotherapy to prevent tumor growth and increase mouse survival. Notably, the enhanced immunotherapy response was found dependent on RIG-I/MDA5-MAVS and cGAS-STING antiviral pathways.

Immunohistochemical analysis and *in silico* evaluation of cancer patient databases showed FBXO44 is frequently overexpressed and correlated with poor prognosis in several major cancer types, including breast, lung, gastric, and ovarian cancers. Furthermore, high FBXO44 expression inversely correlated with DNA replication stress, antiviral pathways, IFN signaling, CD8^+^ T and NK cell infiltration, and tumor antigen presentation in human cancers, corroborating our *in vitro* data and suggesting that targeting FBXO44/SUV39H1 could enhance tumor cell immunogenicity and immunotherapy response. Finally, we generated an immune gene signature related to FBXO44 targeting and demonstrated its ability to predict immunotherapy response in cancer patients.

In summary, our study reported that FBXO44 recruits SUV39H1, CRL4^RBBP4/7^, and Mi-2/NuRD to form a repressive epigenetic complex at the replication fork that promotes H3K9me3-mediated RE silencing post-DNA replication in cancer cells. Functional targeting of FBXO44/SUV39H1 induced DNA replication stress and viral mimicry selectively in cancer cells, leading to inhibition of tumor growth and enhanced anti-PD-1 therapy response in preclinical mouse models. FBXO44/SUV39H1 inhibitors could hold promise as stand-alone cancer therapies or enhancers of onco-immunotherapy response.

